# Fat-to-Muscle Ratio: A New Anthropometric Indicator as a Screening Tool for Metabolic Syndrome in Young Colombian People

**DOI:** 10.3390/nu10081027

**Published:** 2018-08-07

**Authors:** Robinson Ramírez-Vélez, Hugo Alejandro Carrillo, Jorge Enrique Correa-Bautista, Jacqueline Schmidt-RioValle, Emilio González-Jiménez, María Correa-Rodríguez, Katherine González-Ruíz, Antonio García-Hermoso

**Affiliations:** 1 Centro de Estudios Para la Medición de la Actividad Física CEMA, Escuela de Medicina y Ciencias de la Salud, Universidad del Rosario, Bogotá 111221, Colombia; jorge.correa@urosario.edu.co; 2 Grupo GRINDER, Programa de Educación Física y Deportes, Universidad del Valle, Santiago de Cali 76001, Colombia; hugo.carrillo@correounivalle.edu.co; 3 Grupo Interdisciplinario de Estudios en Salud y Sociedad (GIESS), Institución Universitaria Escuela Nacional del Deporte, Santiago de Cali 76001, Colombia; 4 Departamento de Enfermería, Facultad de Ciencias de la Salud, Avda. De la Ilustración, 60, University of Granada, 18016 Granada, Spain; jschmidt@ugr.es (J.S.-R.); emigoji@ugr.es (E.G.-J.); macoro@ugr.es (M.C.-R.); 5 Grupo CTS-436, Adscrito al Centro de Investigación Mente, Cerebro y Comportamiento (CIMCYC), University of Granada, 18071 Granada, Spain; 6 Grupo de Ejercicio Físico y Deportes, Vicerrectoría de Investigaciones, Universidad Manuela Beltrán, Bogotá, DC 110231, Colombia; katherine.gonzalez@docentes.umb.edu.co; 7 Laboratorio de Ciencias de la Actividad Física, el Deporte y la Salud, Facultad de Ciencias Médicas, Universidad de Santiago de Chile, USACH, Santiago 7500618, Chile; antonio.garcia.h@usach.cl

**Keywords:** fat mass, muscle mass, metabolic syndrome, young adults, Latin-Americans

## Abstract

Fat-to-muscle ratio has been proposed as an alternative approach for assessing body fat. The objective of this study was to explore fat-to-muscle ratio thresholds in metabolic syndrome (MetS) diagnosis; it was hypothesised that the fat-to-muscle ratio is a good predictive indicator of MetS in a large population of young Colombian adults. For this purpose, a cross-sectional study was conducted on 1416 subjects (66.6% female), aged from 18.1 to 25.1. As part of the study, measurements of the subjects’ anthropometric indicators, serum lipid indices, blood pressure, and fasting plasma glucose were taken. Body composition was measured through bioelectrical impedance analysis (BIA). A new variable (ratio of fat mass to muscle mass, in kg) was calculated. Following the International Diabetes Federation (IDF) definition, MetS includes three or more metabolic abnormalities. Receiver operating characteristic (ROC) curves and logistic regression determined the discriminatory ability of the fat-to-muscle ratio to predict MetS. According to the IDF, the best fat-to-muscle ratio cut-off point for detecting MetS in men was 0.225 kg, with an area under the curve (AUC) of 0.83, sensitivity of 80%, and specificity of 70%. For women, the fat-to-muscle ratio cut-off point was 0.495 kg, the AUC was 0.88, and the sensitivity and specificity were 82% and 80%, respectively. In conclusion, our results showed that the fat-to-muscle ratio cut-off points from ROC analyses demonstrate good discriminatory power for detecting MetS in young Colombian adults.

## 1. Introduction

Metabolic syndrome (MetS) is a cluster of conditions—raised blood pressure, abnormal cholesterol or triglyceride levels, high blood glucose, and excess body fat around the waist—that occur together, increasing the incidence of type 2 diabetes mellitus, cardiovascular diseases (CVD), and kidney diseases, as well as a greater risk of cardiovascular disease mortality and all-cause mortality [1,2].

According to the 2016-Pan American Health Organisation and Noncommunicable Disease Prevention and Control, MetS has become a serious public health problem [3]. For example, in 2008 the prevalence of MetS in Bogotá, Colombia was 20% for both sexes [4]. Similarly, Davila et al. [5] reported a prevalence of 41% among adults aged 25–64 from Medellin and surrounding municipalities in the Andean Colombian region, and Miranda et al. [6] conducted a cross-sectional study in Armenia, Colombia, of males with a mean age of 45.9 and reported a similar MetS prevalence of 49%, using the definition of the US National Cholesterol Education Programme–Adult Treatment Panel III [7]. For this reason, cardiovascular risk factor measurements are important, even at an early age, for detecting risk profiles in time for intervention [5,8,9].

As adipose tissue plays a central role in MetS, various researchers have begun to explore the predictive ability of anthropometric indicators such as waist circumference (WC), body fat percentage (BF%), body adiposity index, body mass index (BMI), waist-to-height ratio, and fat mass index (FMI) as a screening tool for MetS [10,11]. However, these indicators are not without limitations. For example, BMI provides no data on body composition, and although WC is considered an indirect indicator of visceral fat, repeated training is required to take this measurement accurately and great variability has been reported [12]. On the other hand, anthropometric indicators consider only the effect of fat on metabolism, ignoring skeletal muscle, another relevant tissue.

In fact, skeletal muscle (SM) is the body’s largest non-fat component and can contribute up to 40% of the adult human body weight and be responsible for 30% of energy expenditure [13]. SM has been established as an independent predictor of cardiometabolic diseases and mortality [14,15,16]. Indeed, an inverse association has been reported between SM and the prevalence of MetS in young adults [17,18,19,20], and previous studies have reported that low levels of muscle mass are associated with an increased risk of insulin resistance [21] and CVD [22]. The biological pathways that lead to the protective effect of high SM levels on cardiometabolic health have not yet been determined, but since SM is a primary site for insulin-mediated glucose uptake, loss of SM may promote insulin resistance and, consequently, metabolic disorders [23].

The phenomenon by which SM reduces with the accumulation of adipose tissue is known as sarcopenic obesity, which is associated with significant morbidity [24] and mortality [25]. Xu et al. recently tested other body fat indices with the form fat mass to muscle mass, both in kg, using tetrapolar bioelectrical impedance analyses (BIA), as a surrogate marker of MetS among adults aged 20–80 years from Han and Bouyei populations in China [26]. These authors reported that the fat-to-muscle ratio is highly predictive of MetS and a useful reference indicator. Additionally, Kurinami et al. showed that the muscle-to-fat ratio is also clinically useful for evaluating the presence of insulin resistance [27].

To date, only one previous study has evaluated the applicability of the fat-to-muscle ratio for predicting MetS, with the results confirming its close relationship with the components of MetS [26]. There is, however, no consensus as to the cut-off value that can be used to define excess adiposity based on the fat-to-muscle ratio. Accordingly, the objective of this study was to explore the fat-to-muscle ratio thresholds for diagnosing MetS, under the hypothesis that this ratio is a good predictive indicator of MetS in a large population of young Colombian adults.

## 2. Methods

### 2.1. Study Design

This study is part of the FUPRECOL study [28,29] which included 1,948 Colombian universities. FUPRECOL study is a cross-sectional surveillance study that was carried out between 2014 and 2018, designed to establish the general prevalence of CVD risk factors (anthropometric, adiposity, metabolic, and genetic markers) in the study population (aged 18 to 25.9 years living in Colombia) using a standardized data collection protocol. The study included three data collection sites from seven Colombian universities including Tunja (Boyacá), Cali (Valle del Cauca), and Bogotá (Cundinamarca). The aim was to recruit up to 250 to 500 participants per site over a 4-year data collection period. Ethics approval for this project was originally obtained from the Research Ethics Board (UMB N° 01-1802-2013 and UR N° CEI-ABN026-000262, coordinating centre) and complied with the Declaration of Helsinki (as revised in Hong Kong in 1989 and in Edinburgh, Scotland, in 2000). Each site subsequently obtained technical approval from their respective research board (Bogotá UMB Code N° 01-1802-2013, UR Code N° CEI-ABN026-000010; Cali UNIAJC Code N° 111-02.01.48/16; Tunja Code N° RECT 60). Written informed consent was obtained from all subjects before data collection, and this study also complied with Colombian laws regulating clinical research on human subjects (Resolution 008430/1993 of the Ministry of Health).

### 2.2. Sample Population

Participant recruitment locations were selected across all sites using purposive, non-randomized sampling. Exclusion factors included a clinical diagnosis of CVD, DM-1 and 2, pregnancy, the use of alcohol or drugs, and, in general, the presence of any disease not directly associated with nutrition. Volunteers received no compensation for their participation. Of the 1,934 participants who took part in FUPRECOL study, a total of 1,416 remained in the present analysis after excluding participants without a blood pressure (*n* = 18), body mass index (BMI; *n* = 58), waist circumference (WC; *n* = 29), and metabolic profile (*n* = 204) values. This study used control variables in the analyses, so subjects without information on BIA (*n* = 137) and smoking (*n* = 72) were excluded.

### 2.3. Data Collection Procedures

Data collection staff had a background in CVD risk factors (anthropometric, adiposity, metabolic, and genetic markers) or lifestyle assessment, and were subsequently trained by research staff from the coordinating centre (UR, Bogotá). When measuring body weight, fat mass, and muscle mass, the participants were in light clothing and stood barefoot. Body weight (kg) was measured using an electric scale (Model Tanita^®^ BC-418^®^, Tokyo, Japan) and height (cm) with a portable stadiometer (Seca^®^ 216, Hamburg, Germany). Waist circumference WC (cm) was measured as the narrowest point between the lower costal border and the iliac crest using a metal tape measure (Lufkin W606PM^®^, Parsippany, NJ, USA). BMI and waist-to-height ratio were calculated as weight (kg)/height (m^2^) and WC (cm)/height (cm), respectively. We determined body fat percentage (BF%), fat mass index (FMI) and fat mass (kg) using BIA by whole-body impedance (Tanita Model BC-418^®^, Tokyo, Japan). Detailed information about the BIA technique has been provided in previous studies [30,31]. A new variable, (ratio of fat mass to muscle mass, both in kilograms) was calculated as described in Xu et al. [26]. For all anthropometric variables, a low technical error measurement was reported (less than 2%).

### 2.4. Metabolic Syndrome Diagnosis

Between 6:00 and 9:00 a.m. after 10–12 h of an overnight fast, blood samples were extracted from capillary sampling. Participants were asked not to engage in prolonged exercise 24 h prior to testing. Using enzymatic colorimetric methods, we measured (i) high density lipoprotein cholesterol (HDL-c); (ii) triglycerides (TG); (iii) total cholesterol; and (iv) fasting blood glucose. For all blood samples, we obtained errors of less than 4%.

Blood pressure was measured accurately with an electronic sphygmomanometer (Omron^®^ Healthcare Europe B.V., Hoofddorp, The Netherlands). Individuals were seated in a semi-reclined position with arms relaxed and supported, while the midpoint of the arm was positioned at the level of the heart.

MetS was defined in accordance with the updated harmonized criteria of the International Diabetes Federation (IDF) [32]. According to the criteria of the IDF, participants were considered to have MetS if they showed three or more of the following: (i) central obesity (men with a WC ≥ 90 cm and women with a WC ≥ 80 cm); (ii) increased TG: ≥ 150 mg/dL; (iii) decreased HDL-C level: men < 40 mg/dL and women < 50 mg/dL; (iv) increased systolic blood pressure ≥ 130 or diastolic blood pressure ≥ 85 mmHg; and (v) increased fasting blood-glucose ≥ 100 mg/dL. Thereafter, the TG/HDL and TG/glucose ratios were calculated.

### 2.5. Statistical Analysis

The characteristics of the participants were given as means and standard deviations, or percentages, where appropriate. Independent two-tailed *t*-tests for continuous variables and chi-square (χ^2^) tests for categorical variables were used to examine sex differences.

Receiver-operating characteristics (ROC) were calculated to examine the discriminatory ability of ratio of fat mass to muscle mass to predict MetS by the area under the curve (AUC). AUC has been reported to be a global indicator of diagnostic performance since it represents the ability of the test to correctly classify participants with a high risk of MetS (*p*-values < 0.01 and an AUC > 0.80) [33]. The literature does not provide consensus on what would be the best classifications for AUCs [34,35]. However, AUCs values of 0.55–0.62, 0.63–0.71, and >0.71 corresponded to an effect size (Cohen’s d) of small, medium, and large, respectively [36]. Cutoff points were chosen based on J-Youden index, which uses the point on the ROC parameter that is farthest from the line of equality [37]. In addition, the positive likelihood ratio was also determined.

Finally, an ANOVA was used to investigate whether ratio of fat mass to muscle mass differed by identifying high and low risk of MetS group by applying the ROC cut point in gender groups.

All analyses were performed using the Statistical Package for Social Sciences (IBM-SPSS, version 24.0 for Windows; SPSS Inc., Chicago, IL, USA), and the level of significance was set at alpha < 0.05.

## 3. Results

### 3.1. Study Participants

Table 1 shows the results obtained for the anthropometric, blood pressure, and metabolic biomarker parameters. The final sample comprised a total of 1416 young people. There were 472 males (33.3%) and 944 females (66.6%) with a mean age of 20.8 ± 1.9 (minimum age of 18.1 years and maximum age of 25.1 years). Means (SDs) for the sample were: BMI, 23.5 (3.8) kg/m^2^; WC, 74.2 (9.6) cm; fat-to-muscle ratio, 0.343 (0.163) kg, and 10.3% MetS prevalence using the IDF criteria.

The cohort of women was found to have lower weight, height, WC, waist-to-height ratio, blood pressure, triglycerides, TG/HDL, and TG/G ratios than the men (*p* < 0.001). However, they had higher body fat (% and kg), fat mass index, fat-to-muscle ratio, total cholesterol, LDL, and HDL, in comparison to the men (*p* < 0.001). It is worth highlighting that, in the study sample, the prevalence of MetS was greater in men than women (*p* < 0.001).

### 3.2. Optimal Cut-Off Value in the MetS Screening

The ROC curve analyses for the diagnostic performance of the ratio of fat mass to muscle mass in identifying a high risk of MetS are shown in Table 2. In men, when considering the full sample (*n* = 472), the best fat-to-muscle ratio cut-off point for detecting MetS according to the IDF was 0.225 kg (AUC 0.88, sensitivity 80%, and specificity 70%); for women, the fat-to-muscle ratio cut-off point was 0.495 kg (AUC 0.83, sensitivity 82%, specificity 80%).

### 3.3. Sex Thresholds for High and Low Ratios of Fat Mass to Muscle Mass According to Anthropometric, Blood Pressure, and Metabolic Biomarker Parameters, and Sex

Group- and sex-specific fat-to-muscle ratio thresholds for the presence of MetS according to the IDF, are provided in Table 3, with the corresponding anthropometric, blood pressure, and metabolic profile differences. In both groups the thresholds may, therefore, be used to categorise individuals into two categories of risk (i.e., low and high risk of MetS) on the combined basis of sex and fat-to-muscle ratio group. In men, the optimal cut-off score for the fat-to-muscle ratio showed that there were differences in anthropometric, blood pressure, and metabolic biomarker parameters (all *p* < 0.01).

In women, we found differences in anthropometric, blood pressure, and metabolic biomarker parameters (*p* < 0.001), with the exception of total cholesterol (*p* = 0.161) and LDL-C levels (*p* = 0.209).

## 4. Discussion

Metabolic disturbances, including abdominal adiposity, dyslipidemia, elevated blood pressure, and impaired glucose metabolism, have been identified as risk factors for CVD, as well as all-cause and CVD mortality [1,2]. For this reason, MetS has been established as a worldwide public health problem, with the number of cases rising fastest in Latin-American countries [38]. In our study of a population of young Colombian adults, the total prevalence of MetS was 10.3% using the IDF criteria, with the prevalence being higher in males than females (15.2% vs. 8.0%, respectively). In contrast, in the Cardiovascular Risk Factor Multiple Evaluation in Latin America (CARMELA) study conducted on seven Latin American populations, the estimated prevalence of MetS in Bogotá was 20% [39]. These differences could be explained by the MetS cluster used, since in the CARMELA study MetS was defined according to the National Cholesterol Education Programme Adult Treatment Panel III (NCEP ATP III), in addition to the different age ranges of the two study populations (25–54 vs. 18.1–25.1 years).

Identifying screening tools for predicting MetS early in life is especially relevant in developing effective prevention against MetS in early adulthood. According to Bonomini et al., 2015, in young adults with MetS there is a higher risk of developing insulin resistance, low-grade chronic inflammation and oxidative stress [40]. Therefore, metabolic imbalance at an early age is worrisome, as this may represent the initiation of the atherosclerotic process that is a determinant in the development of CVD [41]. This study provides gender-specific fat-to-muscle ratio reference thresholds for diagnosing MetS in young adults. Our findings showed that the fat-to-muscle ratio has a high discriminatory power for detecting MetS in young Colombian adults, supporting the hypothesis that it is a useful indicator for identifying young people at a high risk of MetS. Taking into consideration the fact that MetS is an important risk factor for CVD incidence [1], these findings are of special interest since they reveal the clinically relevant utility of the fat-to-muscle ratio for detecting metabolic risk in young people.

Fat mass and muscle mass have been independently associated with metabolic risk; however, there was limited evidence on the relationship of the fat-to-muscle ratio with metabolic syndrome [26,42]. To the best of our knowledge, this is the first study to investigate the predictive capacity of fat-to-muscle ratio with respect to MetS in a cohort of young people. The fat-to-muscle ratio thresholds established for detecting a high risk of MetS were ≥0.225 kg in men and ≥0.495 kg in women, with high sensitivity and specificity at the cut-off points for both genders. Furthermore, when comparisons were made by categorising the participants into low and high risk of MetS considering these cut-off points, MetS-related indicators such as anthropometric, blood pressure, and metabolic biomarker parameters in men, and anthropometric, blood pressure, and metabolic biomarker parameters in women were significantly different in the high risk of MetS group compared to the low risk group. This indicates that the cut-off points reported for the fat-to-muscle ratio appear to be useful predictors of metabolic risk in early adulthood.

Our results are consistent with recent findings indicating that fat-to-muscle ratio is a predictor of adult MetS [26,27,42]. In this context, Xu et al. demonstrated that the fat-to-muscle ratio was highly predictive of MetS in a Chinese cohort of 4553 cases of MetS, where the study participants were aged 20–80 [26]. Similarly, a previous study conducted on 61 untreated diabetes mellitus patients found that, in daily clinical practice, fat-to-muscle ratio is clinically useful for evaluating the presence of insulin resistance [27]. Park et al. also reported that the muscle-to-fat ratio indicator was useful in the early prevention and management of MetS in 6256 Koreans subjects [42]. However, differences in ethnicity, age range, and lifestyle of the previous study populations investigated could mean that the results are not directly comparable. Even so, to prevent MetS, it is important to decrease body fat; however, an adequate amount of muscle mass, or an increase in this tissue, is also necessary [21]. This study, in line with the above-mentioned results, supports the importance of promoting physical training to prevent fat mass accumulation and maximise muscle mass to provide metabolic health benefits [43]. The exact mechanisms through which the fat-to-muscle mass ratio determines MetS risk have not been elucidated, but the increased muscle mass might produce metabolic and structural changes that improve muscle insulin sensitivity and glycaemic control [44,45]. Nevertheless, to elucidate the exact mechanisms underlying this association between fat-to-muscle ratio and MetS risk, future longitudinal studies and work involving ethnically different populations are needed.

On the other hand, since body fat mass and muscle mass can be easily measured by bioelectrical impedance analysis, the fat-to-muscle ratio may be used as a powerful predictor of MetS assessed without the need for a full physical examination. Its ease of use makes it a useful complementary assessment measure that could help clinicians prevent MetS and, consequently, the risk of CVD in early adulthood.

This study has potential limitations. Firstly, the cross-sectional design does not allow us to explain causality. For this reason, a future prospective analysis is necessary to determine causal links between the fat-to-muscle ratio and MetS. Secondly, since our study population comprised a well-characterized cohort of young Colombian adults, these findings may not be generalizable to the general population. Therefore, future population-based studies conducted on other ethnic groups and age range are required to provide fat-to-muscle ratio cut-off values for detecting MetS in different populations. Additionally, it also should be noted that we have only applied the IDF criteria to define MetS in our study population. Thus, further studies verifying our results by using also other criteria such as the NCEP-ATP III should be of interest. Finally, although this study was conducted in a large sample size that allows us to provide data on a national scale, further work in larger cohort should be undertaken to confirms our preliminary findings.

Despite these limitations, this is the first research to explore the predictive power of the fat-to-muscle ratio in relation to MetS in a specific population of young adults. Accordingly, this study has relevant clinical implications in that it permits the early detection of MetS at a young age. Additional strengths of this study include the measurements of both fat mass and muscle mass using very reliable measuring devices. Finally, highly standardised procedures have been developed within the FRUPECOL study to avoid measurement bias.

## 5. Conclusions

In summary, our findings support the hypothesis that the fat-to-muscle ratio has a high discriminatory power for detecting MetS in young Colombian adults. This study proposes the first cut-off points for use in clinical practice to predict MetS risk. Considering that MetS in young adults is considered an important public health problem, this study is especially relevant as it provides a novel indicator for the early identification of MetS, which is easy to calculate and suitable for populational level screening. The cut-off points reported can be easily applied to identify populations of young Colombian adults who should be the target of interventions. Future studies of different ethnic groups are required to establish more accurate reference points that are applicable to populations in countries across the globe.

## Figures and Tables

**Table 1:** Characteristics among a sample (mean (standard deviation (SD)) or frequency (%)).

Characteristics	Total (*n* = 1416)	Sex		*p*-Value
		Men (*n* = 472)	Women (*n* = 944)	
**Anthropometric parameters**				
Age, years	20.8 (1.9)	20.6 (2.2)	20.8 (1.7)	0.091
Weight, kg	63.0 (12.6)	71.0 (12.4)	59.0 (10.7)	<0.001
Height, m	1.63 (0.09)	1.72 (0.07)	1.59 (0.06)	<0.001
Body mass index, kg/m^2^	23.5 (3.8)	23.9 (3.6)	23.3 (3.9)	0.008
Waist circumference, cm	74.2 (9.6)	79.6 (9.9)	71.6 (8.3)	<0.001
Waist-to-height ratio	0.455 (0.054)	0.463 (0.056)	0.451 (0.052)	<0.001
Fat mass, kg	15.3 (7.6)	12.5 (6.9)	16.7 (7.5)	<0.001
Body fat, %	23.6 (8.5)	16.8 (6.1)	27.0 (7.3)	<0.001
Fat mass index	5.8 (2.9)	4.2 (2.3)	6.6 (2.8)	<0.001
Fat-to-muscle ratio, kg	0.343 (0.163)	0.218 (0.104)	0.405 (0.151)	<0.001
**Blood pressure**				
Systolic blood pressure	114.8 (12.7)	121.9 (13.0)	111.3 (11.0)	<0.001
Diastolic blood pressure	73.0 (10.0)	75.2 (10.7)	72.0 (9.4)	<0.001
Mean arterial pressure	93.9 (10.0)	98.5 (10.7)	91.7 (8.8)	<0.001
**Metabolic parameters**				
Total cholesterol, mg/dL	141.4 (32.2)	133.4 (29.5)	145.5 (32.8)	0.003
HDL-C, mg/dL	41.6 (11.9)	38.2 (9.9)	43.3 (12.5)	0.018
LDL-C, mg/dL	85.5 (26.2)	80.8 (24.7)	87.7 (26.6)	<0.001
Triglycerides, mg/dL	92.9 (48.0)	99.4 (50.9)	89.7 (46.2)	<0.001
Glucose, mg/dL	87.7 (10.5)	88.4 (9.7)	87.4 (10.8)	0.073
TG/HDL ratio	2.5 (1.6)	2.8 (1.9)	2.3 (1.5)	<0.001
TG/G ratio	8.2 (0.5)	8.3 (0.5)	8.2 (0.4)	<0.001
MetS prevalence, n (%) *	147 (10.3)	72 (15.2)	75 (8.0)	<0.001

Continuous variables are reported as mean values (standard deviations (SDs)) and categorical variables are reported as numbers and percentages in brackets *. Significant between-sex differences (*t*-tests or * chi-square test χ^2^). LDL-C: low-density lipoprotein cholesterol; HDL-C: high-density lipoprotein cholesterol; G: glucose; MetS: metabolic syndrome.

**Table 2:** Diagnostic properties of ratio of fat mass to muscle mass to detect high risk of MetS according to the International Diabetes Federation (IDF) by sex.

Parameter	Sex	
	Men	Women
AUC (Standard error)	0.837 (0.027)	0.889 (0.019)
95% CI	0.782 to 0.892	0.852 to 0.927
*p*-value	<0.0001	<0.0001
J-Youden	0.511	0.627
Cut-off (kg)	0.225	0.495
Sensitivity (95% CI)	0.805 (0.695 to 0.889)	0.826 (0.721 to 0.904)
Specificity (95% CI)	0.706 (0.658 to 0.750)	0.800 (0.772 to 0.827)
Likelihood ratio	2.740	4.153

AUC: area under the curve; CI: confidence interval.

**Table 3:** Sex thresholds of ratio of fat mass to muscle mass to detect high risk of MetS according to the International Diabetes Federation (IDF), for anthropometric, blood pressure, metabolic biomarker parameters, and sex.

Characteristics	Men (*n* = 472)		*p*-Value	Women (*n* = 944)		*p*-Value
	FTMr < 0.225 (*n* = 295)	FTMr ≥ 0.225 (*n* = 177)		FTMr < 0.495 (*n* = 708)	FTMr ≥ 0.495 (*n* = 236)	
**Anthropometric parameters**						
Weight, kg	65.0 (7.3)	81.1 (12.6)	<0.001	54.5 (6.2)	72.8 (9.5)	<0.001
Body mass index, kg/m^2^	21.9 (1.9)	27.2 (3.4)	<0.001	21.7 (2.4)	28.3 (3.3)	<0.001
Waist circumference, cm	74.6 (5.3)	87.9 (10.1)	<0.001	68.3 (5.5)	81.2 (7.4)	<0.001
Waist-to-height ratio	0.434 (0.030)	0.510 (0.057)	<0.001	0.432 (0.038)	0.507 (0.048)	<0.001
Fat mass, kg	8.5 (2.4)	19.0 (7.1)	<0.001	13.3 (4.1)	26.8 (6.3)	<0.001
Body fat, %	13.0 (2.8)	23.0 (5.0)	<0.001	23.9 (5.3)	36.5 (3.6)	<0.001
Fat mass index	1.7 (0.4)	3.7 (1.3)	<0.001	3.3 (1.0)	6.5 (1.4)	<0.001
Fat-to-muscle ratio, kg	0.157 (0.036)	0.321 (0.101)	<0.001	0.337 (0.093)	0.611 (0.099)	<0.001
**Blood pressure**						
Systolic blood pressure	119.4 (12.3)	125.9 (13.1)	<0.001	109.9 (10.8)	115.6 (10.5)	<0.001
Diastolic blood pressure	73.0 (10.1)	78.7 (10.7)	<0.001	71.3 (9.5)	74.1 (9.0)	<0.001
Mean arterial pressure	96.2 (10.1)	102.3 (10.5)	<0.001	90.6 (8.7)	94.9 (8.3)	<0.001
**Metabolic parameters**						
Total cholesterol (mg/dL)	130.3 (26.9)	138.7 (32.9)	0.003	144.6 (33. 3)	148.1 (31.3)	0.161
HDL-C (mg/dL)	39.7 (9.6)	35.7 (9.8)	0.018	44.8 (12.5)	38.6 (11.3)	<0.001
LDL-C (mg/dL)	78.3 (23.2)	84.5 (26.4)	<0.001	87.0 (26.6)	89.2 (6.77)	0.209
Triglycerides (mg/dL)	90.7 (41.7)	113.8 (60.7)	<0.001	84.6 (42.4)	105.1 (53.2	<0.001
Glucose (mg/dL)	87.6 (9.0)	89.8 (10.7)	0.020	86.7 (11.2)	89.3 (9.2)	<0.001
TG/HDL ratio	2.5 (1.6)	3.4 (2.1)	<0.001	2.0 (1.2)	3.0 (1.9)	<0.001
TG/G ratio	8.2 (0.4)	8.4 (0.5)	<0.001	8.1 (0.4)	8.4 (0.4)	<0.001
MetS prevalence, n (%) *	13 (4.4)	59 (33.3)	<0.001	13 (1.8)	62 (26.2)	<0.001

Continuous variables are reported as mean values (standard deviations (SDs)) and categorical variables are reported as numbers and percentages in brackets *. Significant between-sex differences (ANOVA or chi-square test χ^2^). LDL-C: low-density lipoprotein cholesterol; HDL-C: high-density lipoprotein cholesterol; G: glucose; MetS: metabolic syndrome.

## Abbreviations

The following abbreviations are used in this manuscript:

BF	body fat
BIA	bioelectrical impedance analysis
BMI	body mass index
CVD	cardiovascular disease
FUPRECOL	in Spanish: Association between Muscular Strength and Metabolic Risk Factors in Colombia
HDL-C	high-density lipoprotein cholesterol
IDF	International Diabetes Federation
LDL-C	low-density lipoprotein cholesterol
MetS	metabolic syndrome
NCEP ATP III	National Cholesterol Education Program Adult Treatment Panel III
SD	standard deviation
WC	Waist circumference

## References

[1] Mottillo S., Filion K.B., Genest J., Joseph L., Pilote L., Poirier P., Rinfret S., Schiffrin E.L., Eisenberg M.J. (2010). The Metabolic Syndrome and Cardiovascular Risk. J. Am. Coll. Cardiol..

[2] Gami A.S., Witt B.J., Howard D.E., Erwin P.J., Gami L.A., Somers V.K., Montori V.M. (2007). Metabolic Syndrome and Risk of Incident Cardiovascular Events and Death. J. Am. Coll. Cardiol..

[3] Pan American Health Organization (PAHO), Division of Health and Human Development (1995). Health Situation Analysis Program. Health Situation in the Americas: Basic Indicators 1995.

[4] Schargrodsky H., Hernández-Hernández R., Champagne B.M., Silva H., Vinueza R., Silva Ayçaguer L.C., Touboul P.-J., Boissonnet C.P., Escobedo J., Pellegrini F. (2008). CARMELA: Assessment of Cardiovascular Risk in Seven Latin American Cities. Am. J. Med..

[5] Davila E.P., Quintero M.A., Orrego M.L., Ford E.S., Walke H., Arenas M.M., Pratt M. (2013). Prevalence and risk factors for metabolic syndrome in Medellin and surrounding municipalities, Colombia, 2008–2010. Prev. Med. (Baltim).

[6] Medrano M., Maiz E., Maldonado-Martín S., Arenaza L., Rodríguez-Vigil B., Ortega F.B., Ruiz J.R., Larrarte E., Diez-López I., Sarasúa-Miranda A. (2015). The effect of a multidisciplinary intervention program on hepatic adiposity in overweight-obese children: Protocol of the EFIGRO study. Contemp. Clin. Trials.

[7] Expert Panel on Detection, Evaluation, and Treatment of High Blood Cholesterol in Adults (2001). Executive Summary of The Third Report of The National Cholesterol Education Program (NCEP) Expert Panel on Detection, Evaluation, And Treatment of High Blood Cholesterol In Adults (Adult Treatment Panel III). JAMA.

[8] Feliciano-Alfonso J.E., Mendivil C.O., Ariza I.D.S., Pérez C.E. (2010). Cardiovascular risk factors and metabolic syndrome in a population of young students from the National University of Colombia. Rev. Assoc. Med. Bras..

[9] Arteaga-Arredondo L.F., Fajardo-Rodríguez H.A., Fajardo-Rodríguez H.A. (2010). Prevalencia de factores de riesgo cardiovascular en pilotos de aviación civil en Colombia en el año 2005. Rev. Salud Publica.

[10] Sagun G., Oguz A., Karagoz E., Filizer A.T., Tamer G., Mesci B. (2014). Application of alternative anthropometric measurements to predict metabolic syndrome. Clinics.

[11] Mišigoj-Duraković M., Sorić M., Duraković Z. (2014). Anthropometry in cardio-metabolic risk assessment. Arch. Ind. Hyg. Toxicol..

[12] Cornier M.-A., Despres J.-P., Davis N., Grossniklaus D.A., Klein S., Lamarche B., Lopez-Jimenez F., Rao G., St-Onge M.-P., Towfighi A. (2011). Assessing Adiposity: A Scientific Statement From the American Heart Association. Circulation.

[13] Rasmussen B.B., Phillips S.M. (2003). Contractile and nutritional regulation of human muscle growth. Exerc. Sport Sci. Rev..

[14] Roshanravan B., Patel K.V., Fried L.F., Robinson-Cohen C., de Boer I.H., Harris T., Murphy R.A., Satterfield S., Goodpaster B.H., Shlipak M. (2017). Association of Muscle Endurance, Fatigability, and Strength With Functional Limitation and Mortality in the Health Aging and Body Composition Study. J. Gerontol. Ser. A.

[15] Kamada M., Shiroma E.J., Buring J.E., Miyachi M., Lee I. (2017). Strength Training and All-Cause, Cardiovascular Disease, and Cancer Mortality in Older Women: A Cohort Study. J. Am. Heart Assoc..

[16] García-Hermoso A., Cavero-Redondo I., Ramírez-Vélez R., Ruiz J., Ortega F.B., Lee D.-C., Martínez-Vizcaíno V. (2018). Muscular strength as a predictor of all-cause mortality in apparently healthy population: A systematic review and meta-analysis of data from approximately 2 million men and women. Arch. Phys. Med. Rehabil..

[17] Steene-Johannessen J., Anderssen S.A., Kolle E., Andersen L.B. (2009). Low muscle fitness is associated with metabolic risk in youth. Med. Sci. Sports Exerc..

[18] Magnussen C.G., Schmidt M.D., Dwyer T., Venn A. (2012). Muscular fitness and clustered cardiovascular disease risk in Australian youth. Eur. J. Appl. Physiol..

[19] Grontved A., Ried-Larsen M., Moller N.C., Kristensen P.L., Froberg K., Brage S., Andersen L.B. (2015). Muscle strength in youth and cardiovascular risk in young adulthood (the European Youth Heart Study). Br. J. Sports Med..

[20] Fraser B.J., Huynh Q.L., Schmidt M.D., Dwyer T., Venn A.J., Magnussen C.G. (2016). Childhood Muscular Fitness Phenotypes and Adult Metabolic Syndrome. Med. Sci. Sport Exerc..

[21] Srikanthan P., Karlamangla A.S. (2011). Relative Muscle Mass Is Inversely Associated with Insulin Resistance and Prediabetes. Findings from the Third National Health and Nutrition Examination Survey. J. Clin. Endocrinol. Metab..

[22] Kim Y., Han B.-D., Han K., Shin K.E., Lee H., Kim T.R., Cho K.H., Kim D.H., Kim Y.H., Kim H. (2015). Optimal cutoffs for low skeletal muscle mass related to cardiovascular risk in adults: The Korea National Health and Nutrition Examination Survey 2009–2010. Endocrine.

[23] García-Hermoso A., Carrillo H.A., González-Ruíz K., Vivas A., Triana-Reina H.R., Martínez-Torres J., Prieto-Benavidez D.H., Correa-Bautista J.E., Ramos-Sepúlveda J.A., Villa-González E. (2017). Fatness mediates the influence of muscular fitness on metabolic syndrome in Colombian collegiate students. PLoS ONE.

[24] Srikanthan P., Hevener A.L., Karlamangla A.S. (2010). Sarcopenia Exacerbates Obesity-Associated Insulin Resistance and Dysglycemia: Findings from the National Health and Nutrition Examination Survey III. PLoS ONE.

[25] Kim H.-T., Kim H.-J., Ahn H.-Y., Hong Y.-H. (2016). An analysis of age-related loss of skeletal muscle mass and its significance on osteoarthritis in a Korean population. Korean J. Intern. Med..

[26] Xu K., Zhu H.J., Chen S., Chen L., Wang X., Zhang L.Y., Pan L., Wang L., Feng K., Wang K. (2018). Fat-to-muscle Ratio: A New Anthropometric Indicator for Predicting Metabolic Syndrome in the Han and Bouyei Populations from Guizhou Province, China. Biomed. Environ. Sci..

[27] Kurinami N., Sugiyama S., Yoshida A., Hieshima K., Miyamoto F., Kajiwara K., Jinnouchi T., Jinnouchi H. (2016). Correlation of body muscle/fat ratio with insulin sensitivity using hyperinsulinemic-euglycemic clamp in treatment-naïve type 2 diabetes mellitus. Diabetes Res. Clin. Pract..

[28] Ramírez-Vélez R., Correa-Bautista J., Ojeda-Pardo M., Sandoval-Cuellar C., García-Hermoso A., Carrillo H., González-Ruíz K., Prieto-Benavides D., Tordecilla-Sanders A., Martinkėnas A. (2018). Optimal Adherence to a Mediterranean Diet and High Muscular Fitness Are Associated with a Healthier Cardiometabolic Profile in Collegiate Students. Nutrients.

[29] Ramírez-Vélez R., Correa-Bautista J., Sanders-Tordecilla A., Ojeda-Pardo M., Cobo-Mejía E., Castellanos-Vega R., García-Hermoso A., González-Jiménez E., Schmidt-RioValle J., González-Ruíz K. (2017). Percentage of Body Fat and Fat Mass Index as a Screening Tool for Metabolic Syndrome Prediction in Colombian University Students. Nutrients.

[30] Rodríguez-Rodríguez F., Cristi-Montero C., González-Ruíz K., Correa-Bautista J.E., Ramírez-Vélez R. (2016). Bioelectrical Impedance Vector Analysis and Muscular Fitness in Healthy Men. Nutrients.

[31] Ramírez-Vélez R., Correa-Bautista J., Martínez-Torres J., González-Ruíz K., González-Jiménez E., Schmidt-RioValle J., Garcia-Hermoso A. (2016). Performance of Two Bioelectrical Impedance Analyses in the Diagnosis of Overweight and Obesity in Children and Adolescents: The FUPRECOL Study. Nutrients.

[32] Alberti K.G.M.M., Eckel R.H., Grundy S.M., Zimmet P.Z., Cleeman J.I., Donato K.A., Fruchart J.-C., James W.P.T., Loria C.M., Smith S.C. (2009). Harmonizing the Metabolic Syndrome: A Joint Interim Statement of the International Diabetes Federation Task Force on Epidemiology and Prevention; National Heart, Lung, and Blood Institute; American Heart Association; World Heart Federation; International Atherosclerosis Society; and International Association for the Study of Obesity. Circulation.

[33] Bewick V., Cheek L., Ball J. (2004). Statistics review 13: Receiver operating characteristic curves. Crit. Care.

[34] Craig E., Bland R., Ndirangu J., Reilly J.J. (2014). Use of mid-upper arm circumference for determining overweight and overfatness in children and adolescents. Arch. Dis. Child.

[35] Rice M.E., Harris G.T. (2005). Comparing effect sizes in follow-up studies: ROC Area, Cohen’s d, and r. Law Hum. Behav..

[36] DeLong E.R., DeLong D.M., Clarke-Pearson D.L. (1988). Comparing the areas under two or more correlated receiver operating characteristic curves: A nonparametric approach. Biometrics.

[37] Fluss R., Faraggi D., Reiser B. (2005). Estimation of the Youden Index and its associated cutoff point. Biom. J..

[38] Roth G.A., Forouzanfar M.H., Moran A.E., Barber R., Nguyen G., Feigin V.L., Naghavi M., Mensah G.A., Murray C.J.L. (2015). Demographic and Epidemiologic Drivers of Global Cardiovascular Mortality. N. Engl. J. Med..

[39] Escobedo J., Schargrodsky H., Champagne B., Silva H., Boissonnet C.P., Vinueza R., Torres M., Hernandez R., Wilson E. (2009). Prevalence of the Metabolic Syndrome in Latin America and its association with sub-clinical carotid atherosclerosis: The CARMELA cross sectional study. Cardiovasc. Diabetol..

[40] Bonomini F., Rodella L.F., Rezzani R. (2015). Metabolic Syndrome, Aging and Involvement of Oxidative Stress. Aging Dis..

[41] Berenson G.S., Srinivasan S.R., Bao W., Newman W.P., Tracy R.E., Wattigney W.A. (1998). Association between Multiple Cardiovascular Risk Factors and Atherosclerosis in Children and Young Adults. N. Engl. J. Med..

[42] Park J., Kim S. (2016). Validity of muscle-to-fat ratio as a predictor of adult metabolic syndrome. J. Phys. Ther. Sci..

[43] World Health Organization (2010). Global Recommendations on Physical Activity for Health.

[44] Klimcakova E., Polak J., Moro C., Hejnova J., Majercik M., Viguerie N., Berlan M., Langin D., Stich V. (2006). Dynamic Strength Training Improves Insulin Sensitivity without Altering Plasma Levels and Gene Expression of Adipokines in Subcutaneous Adipose Tissue in Obese Men. J. Clin. Endocrinol. Metab..

[45] Holten M.K., Zacho M., Gaster M., Juel C., Wojtaszewski J.F.P., Dela F. (2004). Strength training increases insulin-mediated glucose uptake, GLUT4 content, and insulin signaling in skeletal muscle in patients with type 2 diabetes. Diabetes.

